# Use of photography to identify free-roaming dogs during sight-resight surveys: Impacts on estimates of population size and vaccination coverage, Haiti 2016

**DOI:** 10.1016/j.jvacx.2019.100025

**Published:** 2019-06-19

**Authors:** Julie M. Cleaton, Jesse D. Blanton, Pierre Dilius, Fleurinord Ludder, Kelly Crowdis, Alexandra Medley, Richard Chipman, Frantzlet Estime, Emanuel Maciel, Ryan M. Wallace

**Affiliations:** a Poxvirus and Rabies Branch, Division of High Consequence Pathogens and Pathology, National Center for Emerging and Zoonotic Infectious Diseases, Centers for Disease Control and Prevention, Atlanta, GA, USA; b Direction Production et Santé Animale/Protection Sanitaire, Ministère de l’Agriculture, des Ressources Naturelles et du Développement Rural, Port-au-Prince, Haiti; c Christian Veterinary Mission, Port-au-Prince, Haiti; d Quarantine and Border Health Services Branch, Division of High Consequence Pathogens and Pathology, National Center for Emerging and Zoonotic Infectious Diseases, Centers for Disease Control and Prevention, Atlanta, GA, USA; e USDA, APHIS, Wildlife Services National Rabies Management Program, Concord, NH, USA; f Humane Society International, Washington, DC, USA

**Keywords:** Dog population estimation, Dog vaccination, Free-roaming dogs, Free-ranging dogs, Capture-recapture, Sight-resight, Mark-resight

## Abstract

**Background:**

Enumerating dog populations is essential to plan and evaluate rabies vaccination campaigns. To estimate vaccination coverage and dog population size in a Haitian commune, 15 sight-resight counts were conducted over two days following a government-sponsored vaccination campaign.

**Methods:**

Dogs received temporary laminated collars and livestock wax marks on the head and sides at the time of rabies vaccination. After the vaccination campaign, pairs of surveyors walked pre-defined routes through targeted neighborhoods, photographing and recording characteristics and location of each dog seen on a standardized data sheet. On the second survey day, surveyors retraced the prior day’s track, followed the same procedure, and indicated in addition whether they believed the dogs were resighted from the prior day. After completion of the field survey, two independent evaluators reviewed photographs and characteristics of each dog to assess which had vaccination marks and which were resighted. Surveyor and photo-reviewer sight-resight decisions were compared using Cohen’s kappa, and population estimates were compared using Lincoln-Petersen 95% confidence intervals.

**Results:**

Field-surveyors identified dogs consistent with the photograph evaluations in 629 out of 800 instances (78.6%, Cohen’s kappa of 0.12). Despite this inconsistency, the population estimates resulting from the field and final determinations were not significantly different at 1,789 (95% CI 1,677 to 1,901) and 1,978 (95% CI 1,839 to 2,118). Vaccination coverage was also the same at 55% and 56%; however, an observed vaccination mark loss of 13.8% suggests that the true coverage may have been closer to 64%.

**Conclusion:**

Using photos improved dog identification during the sight-resight study, leading to a higher population estimate. Despite using a 2-mark system to temporarily identify vaccinated dogs, a significant proportion had lost all identifying marks by the second day of field surveys. Efforts to estimate vaccination coverage using sight-resight surveys should consider improvement of marking techniques or better accounting for potential loss of marks in their free-roaming dog vaccination coverage assessments.

## Highlights

• Reviewing photographs helped identify dogs seen on both survey days.
• Improved dog identification lead to a higher population estimate.
• Vaccination coverage did not vary between field-surveyor and photo-reviewer populations.
• Improved dog identification allowed for vaccination mark loss estimates, showing likely higher coverage.

## 1 Introduction

Rabies and other zoonotic conditions often afflict free-roaming dog populations. An estimated 59,000 people die from rabies every year, and the vast majority of those cases come from the bite of an infected dog [1]. In Haiti there are an estimated 130 human deaths due to rabies each year [1]. In order to implement and evaluate rabies control measures, it is important to have reliable estimates of the dog population. Various methods exist for estimating animal population sizes, from simple counts and surveys to advanced models, but few are better suited to free-roaming animals than capture-recapture methods [2], [3]. For dogs, this method requires all visible animals in a defined area to be captured and marked (or in the case of mark-resight, not captured but marked with a sprayed dye) on day one and then document the number recaptured the following day to record the proportion of marked to unmarked animals. This proportion is then used to extrapolate to the estimated total population within the representative area [4].

One limitation of the capture-recapture method is that capture procedures and marking techniques may scare off or traumatize the targeted animals, resulting in their fleeing the area or hiding from surveyors on day 1 or when recapture attempts are conducted the next day. A mark-resight study in Thailand found that 12% of dogs seen were too afraid or aggressive to be marked on day 1 of the study, so those would not have been counted as resights if observed again on day 2 [5]. A similar but potentially less biased method, sight-resight (SRS), has been used in which surveyor recall and recorded physical characteristics of the dogs are used in lieu of a mark to determine if a dog was resighted (or “recaptured”). This SRS method has been noted to be practical in low-income countries due to its low cost and simplicity [2]. However, concerns have been raised about the ability of surveyors to accurately recall and identify individual dogs, particularly in communities where dogs have very similar physical features [6]. Photography has been proposed as one means of improving accuracy of unique dog identification [6].

In addition to using SRS surveys to census dog populations, these methods have also been used to estimate post-campaign vaccination coverage. Vaccination coverage can be estimated through application of a physical mark on vaccinated dogs, which is then recorded during the post-vaccination SRS survey. However, temporary vaccination marks (i.e. temporary collars, livestock wax marker crayon, or sprayed dyes) may be lost shortly after vaccination or may not be easily visible to surveyors. One study reported that 6.8% of owned, vaccinated dogs had lost their vaccination collars within just two days of the campaign [7]. If post-vaccination SRS were used to estimate free-roaming dog vaccination coverage, loss of mark could lead to inaccurate coverage estimates. In this SRS study, surveyors provided their field-based determination of resighted dogs and took photographs that were later analyzed by independent reviewers in an attempt to improve the accuracy of population and vaccination coverage estimates.

## 2 Methods

In August 2016, Haiti’s Ministry of Agriculture, Natural Resources and Rural Development (MARNDR) in conjunction with the US Centers for Disease Control and Prevention (CDC) and Christian Veterinary Mission (CVM) conducted an evaluation of a government-sponsored vaccination campaign in the commune of Croix-des-Bouquets, Ouest Department. Five sites were randomly selected within Croix-des-Bouquets to compare vaccination methods and three 2-day post-vaccination SRS surveys were conducted at each site (a total of 15 SRS surveys). The five study sites were defined based on estimated human population, with each zone containing approximately equivalent human populations (7,200 people and 800 dogs per zone). Vaccination campaigns were conducted over a two-day period in three urban sites and two less developed peri-urban sites. Vaccinators were employed by MARNDR and had at least two years of vaccination campaign experience. Vaccinators applied two marks to every vaccinated dog: livestock wax marks on the head and at least one side of each dog and TabBand© laminated collars [8].

SRS surveys took place the day after completion of the 2-day vaccination campaign. SRS surveyors were veterinary agents who had completed a two-year agricultural post-graduate training program and were recommended by MARNDR for their advanced skills with electronic equipment and familiarity with the community in which the SRS took place. SRS surveyors underwent a 4-day training on SRS methodology, using the Global Positioning System (GPS) and digital camera device, and the study methodology prior to conducting the vaccination evaluation. Training was conducted in Haitian Creole, the local and preferred language of the SRS surveyors. Pairs of SRS surveyors walked for approximately five kilometers along a standardized route for two consecutive days, during the hours of 2 pm – 5 pm. Garmin Montana 680 GPS units with 8 megapixel digital cameras were used to track the paths traversed by the surveyors and the location of all dogs seen over the survey period. Photographs and standardized data were captured for each dog sighted on each day. Information collected included age, sex, color, body condition score, wounds, location of sighting, presence of a vaccination mark, the geotag number for the photograph, and on day two whether they believed that they had seen the dog the previous day (Appendix A). Forms and training materials were translated into Haitian Creole by MARNDR staff. Field determination of resight was based on surveyor recall.

After completion of field-data collection, the forms were transcribed into an excel database and each dog was given a unique identification number. Two reviewers independently evaluated photographs to identify dogs sighted on both SRS days 1 and 2, hereby referred to as “resighted dogs.” Photo-reviewers were blinded to the field surveyors’ resight determinations during the initial review process. Digital photographs were viewed on desktop computers, comparing side by side each day 1 photo to all the day 2 photos for that site. Dogs for which a match was identified were considered “resights” and were given a shared identification number for each day, and those for which there was no match were declared “sights.” When photographs were unavailable or unclear, photo-reviewers considered those dogs sights until later comparing with the field surveyors’ decisions.

**Fig. 1:**
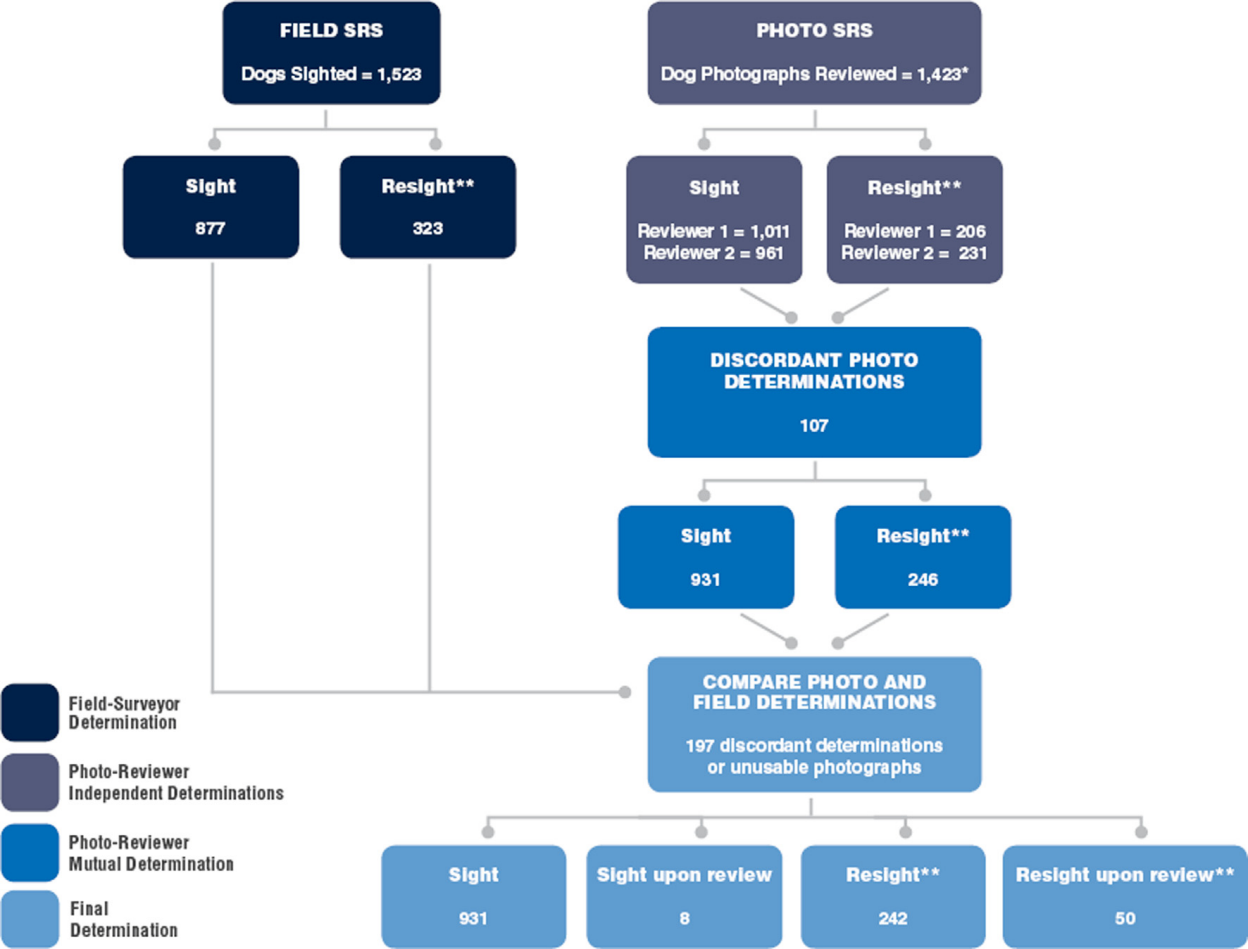
Flowchart of the SRS review and decision process. *Due to poor image quality or upload failure, 100 fewer photos were available than dogs sighted. While those could not be judged as sights or resights by the photo-reviewers, they were later accounted for in the review process. Non-photographed dogs were determined to be resights when the field-surveyors named them so and there was a dog with a matching description from the previous day. **The resights are only counted as one dog in this flowchart, so to reach the total numbers reviewed they must be doubled.

Results between the two independent photo-reviewers were compared for consistency in identifying each dog as a sight or resight. When there was discordance between photo-reviewers, the photographic evidence as well as field data were re-examined and reviewers made a consensus determination. After photo-reviewer independent and consensus determinations on dog identifications were finalized, results were compared to field-surveyor determinations. Discordant results were again assessed, considering all photographic and form documentation, and the photo-reviewers made a final determination on if they were resighted. Dogs with missing or poor quality photos were initially called sights, and descriptions of the dogs were used to find potential matches if the field surveyors called them resights. New sights and resights from this step were named sights and resights upon review (Fig. 1). This final determination was considered the gold standard for comparison to the field surveyor and initial photo reviewer decisions. The independent photo reviewers, photo review consensus, field-surveyor and final determinations were compared for concordance using Cohen’s kappa coefficients calculated in Excel.

Resighted dogs’ data were analyzed to estimate the final proportion vaccinated and to examine collar loss and wax mark loss (referred to collectively as mark-loss). Collar and wax loss were calculated using SAS 9.4. Mark-loss was calculated over a four-day period: Time period one (TP-1) was from the time of vaccination to the first day of the SRS survey (1–2 days depending on when the dog was vaccinated). Time period two (TP-2) was the time from the first day of the SRS to the second SRS day (1 day). To estimate collar and wax mark loss during TP-1, the following calculation was performed for two dog populations; those that were only seen once (“sighted”) and those that were seen on both SRS day 1 and 2 (“resighted”):

$$\mathrm{Collar}\;Loss=D_{w}/\left(D_{w}+D_{c}+D_{\mathrm{wc}}\right)$$

$$\mathrm{Wax}\;Loss=D_{c}/\left(D_{w}+D_{c}+D_{\mathrm{wc}}\right)$$

where *D_w_* are dogs with only wax marks, *D_c_* are dogs with only collars and *D_wc_* are dogs with both wax marks and vaccination collars (Table 3, Table 4). Total mark loss (both wax and collar loss) were also estimated among resighted dogs by recording dogs that had at least one mark during SRS day one and no marks on SRS day two. Collar loss and wax mark loss estimates were plotted by time period and linear, logarithmic, and exponential models were evaluated; models with the highest R-square goodness of fit value are reported in Fig. 4.

Two methods were conducted to evaluate field surveyor’s ability to recognize and record the presence of a vaccination mark (collar or wax). First, the accuracy of recording vaccination marks on the paper record was evaluated by comparing paper records to a 10% subset of the photographs. It was pre-determined that if discordance between photographic and paper records exceeded 2.5% then all photos would be reviewed for mark identification. Second, paper records were evaluated among all resighted dogs to determine if marks were identified on day 2 that were not identified on day 1 (referred to as mark misidentification). To estimate mark misidentification, the number of resighted dogs recorded as marked or collared on the second day but not the first was divided by the number of eligible vaccinated dogs.

## 3 Results

There were 1,523 dog sightings recorded by field surveyors across the 15 survey locations, and photos were available for 93.4% of these. One SRS location was erroneously surveyed prior to vaccination; therefore, this data was not included in vaccination coverage or collar and wax mark analysis. A total of 15 SRS surveys were considered for evaluating dog identification and impact on population estimates. A total of 14 SRS surveys were considered for evaluating vaccination coverage and collar and wax mark loss.

**Fig. 2:**
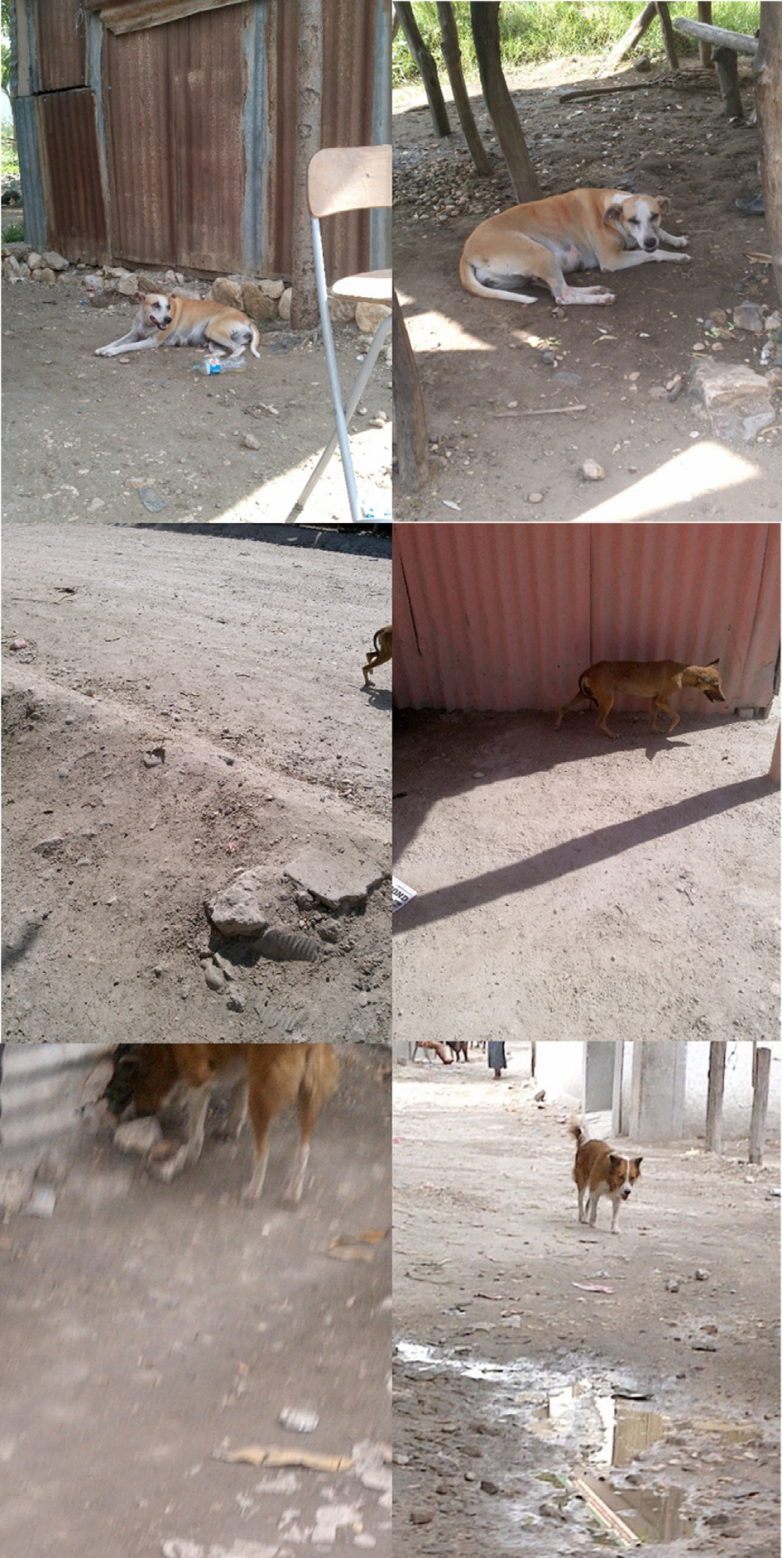
Examples of resights in varying categories. The upper left and upper right photos were a concordant resight between photo-reviewers. This dog is also an example in which the wax vaccination mark was visible to field-surveyors both days, but only to photo-reviewers on the second day. The middle left and right photos were a discordant resight between photo-reviewers. The bottom left and right photos were initially called a sight by the photo-reviewers. After revealing the field-surveyors’ decisions and re-examining the photos with discordant decisions, this dog was called a resight upon review.

Of the 292 dogs with the final determination of resight, field surveyors correctly identified 222 (76.0%) (Table 1). Field surveyors incorrectly identified 101 dogs as resights when compared to the final determination. Field surveyors failed to identify 70 dogs identified as resights per final determination. The consensus determination of the photo-reviewers resulted in resight identifications consistent with the final determination in 242 of the 246 dogs they identified as resights (98.4%; 82.9% of final determination) (see Table 2). Photo-reviewer consensus determination did not identify 50 (17.1%) resighted dogs, based upon the final determination (Fig. 2).

**Table 1:** Observed resights and proportion consistent with the final determinations with 95% confidence intervals across 15 sites.

Site	Counts		Field-surveyor determined resighted dogs			Photo-reviewer mutually determined resighted dogs			Final determination of resighted dogs
	SRS Count 1	SRS Count 2	Reported	Consistent (%)	95% CI	Reported	Consistent (%)	95% CI	
Urban (n = 9, 875 photos)	459	475	186	129 (69.4%)	58–82%	127	124 (97.6%)	81–100%	162
Peri-Urban (n = 6, 548 photos)	264	325	137	93 (67.9%)	55–83%	119	118 (99.2%)	82–100%	130
Total (n = 15, 1,423 photos)	723	800	323	222 (68.7%)	60–78%	246	242 (98.4%)	87–100%	292

**Table 2:** Population estimates from field and photo reviews across 15 sites. Bolded numbers indicate values outside the 95% confidence intervals for the final estimates.

Site	Counts		Field-surveyor determination		Photo-reviewer mutual determination*		Final determination		
	SRS Day 1	SRS Day 2	Resight	Dog Population Estimate	Resight	Dog Population Estimate	Resight	Dog Population Estimate	95% CI
Urban (n = 9, 875 photos)	459	475	186	1,170	127	**1,710**	162	1,342	1,208–1,477
Peri-Urban (n = 6, 548 photos)	264	325	137	625	119	719	130	658	596–720
Total (n = 15, 1,423 photos)	723	800	323	1,789	246	**2,347**	292	1,978	1,839–2,118

* When photographs were unavailable or unclear, photo-reviewers considered those dogs sights until comparing with the field surveyors’ decisions.

Field surveyors reported 323 resighted dogs (25.7% of unique dogs), which resulted in a Lincoln-Petersen population estimate of 1,789 dogs (95% CI 1,677 to 1,901). Photo-reviewers mutually determined there were 246 resighted dogs (18.3% of unique dogs), which resulted in a Lincoln-Petersen population estimate of 2,347 dogs (95% CI 2,235 to 2,459; 31.2% more dogs than the field-surveyor determination). Final determination identified 292 dogs as resights (22.6% of unique dogs), leading to a free-roaming dog population estimate of 1,978 (CI 1,839–2,118), a 10.6% increase from field surveyor estimates and a 15.7% reduction from the photo-reviewer estimates (Table 1). Twenty-four (8.2%) of these dogs were resights upon review because of missing or low quality photos.

The concordance for unique dog identification between photo-reviewers A and B (photo-reviewer independent determination) was moderate with a Cohen’s kappa value of 0.46, while the concordance between the field surveyor’s determination and the photo-reviewer’s mutual determination was poor at 0.12. The field surveyor and photo reviewer concordances to the final SRS determination were 0.30 and 0.75, respectively.

Among the subset of 309 photographs and records reviewed to determine accuracy of field surveyor identification of wax and collar presence, the field surveyors missed only one wax mark (0.3%) and no collars (0%) based on what the photo reviewer could confirm by digital dog photography. On the other hand, the field surveyors could observe marks much more effectively than the photo reviewers could. Out of 137 dogs recorded to have a collar, only 107 (78.1%) collars were visible in the corresponding photos due to distance or angle. Out of 167 dogs recorded to have a wax mark, only 33 (19.8%) were visibly marked in the photos. The field surveyors’ observations were used for coverage estimates, as they were better able to distinguish the marks in person.

The following results combine the photo-reviewed SRS data with the field surveyors’ vaccination mark observations. Among the 14 SRS sites eligible, a total of 899 unique dogs were sighted (Table 4). A total of 411 unique dogs were sighted on SRS day one, of which 238 had a mark of vaccination (collar and/or wax) (57.9%), and 488 unique dogs were sighted on SRS day 2 of which 251 had a mark of vaccination (51.4%). On SRS day one, 123 of 238 (51.7%) vaccinated dogs had both collar and wax marks, 193 (81.1%) had a wax mark and 168 (70.6%) had a collar; 45 dogs had a collar but had lost the wax mark (18.9%) and 70 dogs had a wax mark but had lost the collar (29.4%). On SRS day two, 119 of 251 (47.4%) vaccinated dogs had both collar and wax marks, 189 (75.3%) had a wax mark and 181 (72.1%) had a collar; 62 had a collar but had lost the wax mark (24.7%) and 70 had a wax mark but had lost the collar (27.9%). Complete mark-loss could not be determined among dogs sighted on only one day.

**Table 3:** Vaccination coverage estimates with mid-P exact 95% confidence intervals based on the three sets of sights and resights, and the final estimate inflated by 13.8% to compensate for vaccination mark loss.

	Field-surveyor determination (%, 95% CI)	Photo-reviewer mutual determination	Final determination	Final plus mark loss correction
Urban	51 (48–55)	53 (49–56)	53 (49–56)	60 (56–63)
Peri-Urban*	62 (57–66)	63 (59–68)	64 (59–68)	73 (68–77)
Total*	55 (52–58)	56 (54–59)	56 (54–59)	64 (62–67)

* Vaccination coverages exclude one peri-urban site where the SRS was not conducted in the vaccination area.

**Table 4:** Estimated mark loss among the 899 sights (excluding resighted dogs from each day) across 14 sites.

	Time period 1 (n = 411)		Time period 2 (n = 488)	
Total marked	238 (57.9% coverage)		251 (51.4% coverage)	
Wax Lost	45	18.9%	62	24.7%
Collar Lost	70	29.4%	70	27.9%
Both Lost	Unknown		Unknown	

**Fig. 3:**
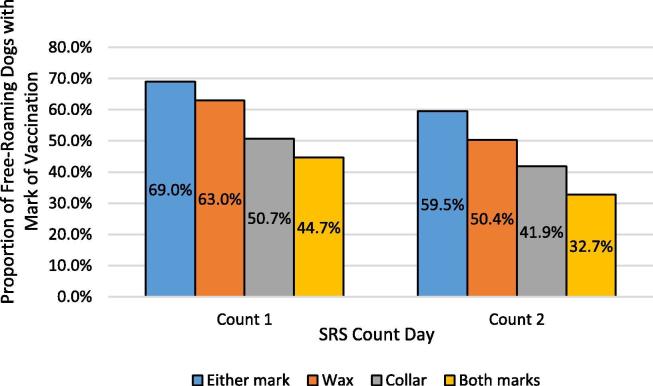
Apparent Vaccination Coverage among Resighted Dogs. Vaccination coverages among 284 resighted dogs by mark type, excluding one *peri*-urban site in which the SRS was not conducted in the vaccination area. *Mid-P exact, 1-tailed p-values were calculated for the difference in observed coverage for each marking method and assessed at the alpha = 0.05 level. Loss of either mark was insignificant between the two count-days (0.08). Loss of wax mark was significant (0.02). Loss of collar was not significant (0.06). Loss of both marks was significant (0.01).*

**Fig. 4:**
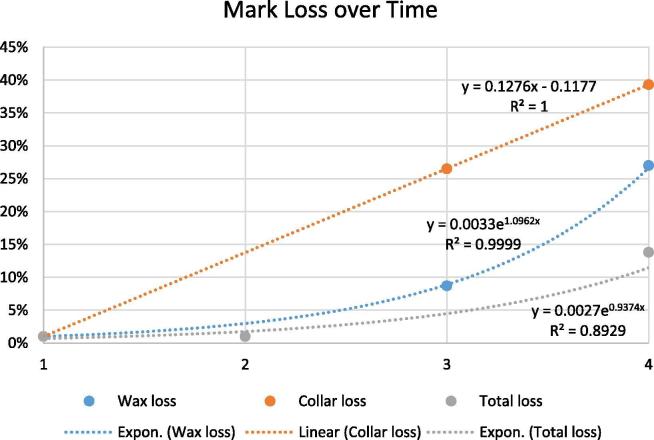
Loss of collar and wax marks over a 4-day period post-vaccination.

Among the 292 dogs that were classified as resights by final determination, 196 had evidence of vaccination (wax, collar, or both). For 17 dogs, vaccination marks were not observed on SRS day one (5.8%), but were observed on SRS day two; for the purposes of analysis, these dogs were considered to have had these marks on day 1. Vaccination coverage among resights, as determined by presence of either mark, on SRS days one and two were 69.0% and 59.5% (Fig. 3). On SRS day one, 8.7% of the 196 vaccinated, resighted dogs had lost the wax mark and 26.5% had lost their collar (Table 5). By SRS day 2, 27.0% of vaccinated, resighted dogs had lost their wax mark and 39.3% had lost their collar. The proportion of vaccinated, resighted dogs that had lost both marks as of SRS day two, resulting in no observable mark of vaccination, was 13.8%. No dogs with both wax and a collar present on SRS day 1 had lost both at the time of the second count. Cumulative collar loss rates over the four day period were best characterized by a linear trend line (equation: y = 0.1276x − 0.1177, R^2^ = 1.0) (Fig. 4). Cumulative wax loss rates over the four day period were best characterized by an exponential trend line (equation: y = 0.0033e^1.0962x^, R^2^ = 0.999). Cumulative total mark loss over the four day period was best characterized by an exponential trend line (equation: y = 0.0027e^0.9374x^, R^2^ = 0.893).

**Table 5:** Cumulative mark loss among the 284 resighted dogs across 14 sites.

	Time period 1		Time period 2	
Total Marked	196 (69.0% coverage)			
Wax Lost	17	8.7%	53	27.0%
Collar Lost	52	26.5%	77	39.3%
Both Lost	Unknown		27	13.8%

## 4 Discussion

Methods for estimating post-vaccination coverage in dog populations that are largely free-roaming are fraught with error [9]. Practices that improve identification of vaccinated dogs, perhaps through digital photographs or improved marking methods, may reduce this error. At a minimum, understanding the limitations of these identification and marking methods can help to refine coverage estimates, particularly when reported vaccination coverages are not followed by expected declines in rabies cases. In this vaccination evaluation, use of digital photography was useful to validate reports from field surveyors and more accurately identify individual dogs to enhance population estimates. This method likely improved dog identification, allowing compensation for loss of marks, and presumptively improved vaccination coverage and population estimates. Field surveyors did not have high concordance with the photo-reviewers in terms of identifying resighted dogs, which could have led to inaccuracies in dog population estimates. However, photographs alone are unlikely to capture usable images of all dogs and as such it is possible that resighted dogs can be missed without also considering field data. One site had to be excluded from the study because all its photos were lost, a limitation for both digital and physical data. Additionally when estimating vaccination coverage, the records of wax marks and collars from field surveyors were more reliable than using photographs alone. Without highly visible marks from all angles, field recordings of marks will remain essential for estimating vaccination coverage. The two-mark system and photo resight identification allowed for probable improvements to the accuracy of vaccination coverage estimates among free-roaming dogs. The photo-based method is time-consuming, but these results suggest that it reliably improves accuracy of dog identification, which would refine and enhance population and vaccination coverage estimates. In future enumeration activities (particularly if they will involve numerous communities such as during a national vaccination campaign), the use of crowdsourcing technologies should be explored to distribute the time burden to review photos [10].

### 4.1 Enumerating free-roaming dog populations

The Lincoln-Petersen method of population estimation is based upon the fraction of dogs that are declared resights, therefore accurate determination of this proportion is critical. Field surveyors were less accurate at identifying unique, individual dogs; and hence field teams had lower accuracy in identifying resighted dogs when compared to photo reviewers. Of the resighted dogs identified by field and photo teams, only 68.7% of the field surveyor’s determinations matched the final determination, compared to 98.4% match-rate for the photo reviewers. Furthermore, field-surveyors failed to identify 70 dogs that were determined to be resights, compared to only 50 failures for the photo team. However, photographs were not available or not useful for identification due to poor quality for approximately 7% of the dogs, so the field surveyors’ written information was helpful in making the final determination for dog identification. Field data was also critical to come to a mutual determination when photo-reviewers were in disagreement. While field-surveyors may be prone to error in unique dog identification during the SRS method, information collected from the field can improve accuracy of dog identification when considered in addition to a secondary method of confirmation such as photography.

Tools to help field surveyors correctly identify dogs, and thereby eliminate the need for post-survey photographic review, would greatly aid in the ability to accurately estimate the size of free-roaming dog populations. The non-governmental agency Mission Rabies has published on a mobile phone application that is used for just these purposes [11]. Further development and validation of tools such as these is necessary to understand the benefits and disadvantages of such technology. In Haiti, numerous free roaming dogs are often seen roaming in packs. It may not be feasible to rely on a rigid survey tool in which each dog is logged individually, whereas paper forms enable the recording of information on multiple animals simultaneously. Additionally, use of mobile data collection devices could add significant delays if field surveyors are expected to review previous day’s records to decide if each dog was already sighted. The accuracy of the Lincoln-Petersen method is reliant upon an adequate sample size; too few dogs sighted and the population estimates are unreliable. Therefore, adopting new technology that aids in resight accuracy must balance this with other practical considerations including the additional time required to complete surveys.

### 4.2 Assessing vaccination coverage

Field surveyors were better able to see marks of vaccination than photo reviewers, so in this respect field observations and paper records are critical. Comparing photographs to field observations related to vaccination marks allowed for the determination of mark-loss rates, and the ability to adjust vaccination coverages accordingly. Among dogs with one mark on the first count, 13.8% had lost that mark after just one day and would not have been considered vaccinated without the photo review. These findings highlight the importance of using more than one marking method to obtain accurate vaccination coverage estimates in free-roaming dogs. Alternatively, highly visible and long-lasting marking methods should be considered. However, we are not aware of any temporary marking methods that are low-cost, easily applied, highly visible, and long-lasting.

Identification of marks of vaccination can be heavily influenced by the experience of the surveyors, marking methods, density of dogs in the area, community behaviors, and other factors. The rate of mark-loss reported here may not be representative of all vaccination settings, but these findings do highlight that mark-loss can have a significant impact on interpretation of the post-vaccination immunization rates (Fig. 4). While digital photography can be helpful to characterize the mark-loss rate for a vaccination program, the intensive effort to capture photographs and independently review should be weighed against their marginal benefits beyond what field data provides. Future evaluations may consider using photography for a subset of the study population, the results of which could be used to provide confidence in the overall estimates.

Reports from field vaccinators suggested that owners removed collars from their dogs shortly after vaccination, likely due to misunderstanding regarding the collar’s purpose. Field vaccinators also reported that some dogs removed the collars shortly after placement by scratching them. Better education of owners on the purpose of the collar and use of stronger collars may improve mark retention. However, anything that takes more time to apply to the dog will slow the vaccination teams and result in potentially lower coverage. The cost of marking vaccinated dogs must also be considered, as many canine rabies endemic countries struggle to identify adequate funding for mass rabies vaccination campaigns, and these marking costs are likely to be incurred by the vaccination program. Wax markings followed an exponential trend and faded away at an increasing rate over time. Dogs and owners did not seem bothered by the wax markings, reducing the immediate removal as reported with collars, but they can easily become less visible due to rain, dirt, and decay. Findings from this evaluation support the use of two marking methods for identifying vaccinated dogs, and highlight the need for more durable, low-cost marking materials.

Loss and visibility of a vaccination mark is a major concern when trying to estimate coverage with an SRS survey, and in this evaluation we have shown that even with a 2-mark method the impact can be significant. In this study we found that as many as 6% of marked dogs may be misclassified by survey teams, presumably due to their inability to see the mark. This may happen when a wax mark is placed on only one side of a dog, or when a collar is hidden by fur. It is also almost assuredly related to how close the surveyor can get to the dog and the amount of time the surveyor has to evaluate the dog.

Mark-loss was directly related to the duration between mark-application and the SRS survey. Just one to two days after placement, more than 10% of marks were lost or not visible. Between the second and third day after placement, over 15% of marks were no longer visible. These models describing trends in mark-loss over time indicate that even minor delays of several days between marking dogs and conducting the SRS can have large impacts on the coverage estimates, and the rate of mark-loss can differ widely between marking method (Fig. 4). Therefore, immediately conducting the SRS is important to evaluate coverage. If vaccination is drawn out over multiple days, you will likely under-estimate the free-roaming dog vaccination coverage when using SRS.

Field surveyors, when relying upon memory recall, may not accurately identify unique dogs. This could result in spurious population estimates, especially if counts are conducted over more than two days. However, field surveyors are more likely to accurately identify marks of vaccination compared to a photo-based method. When determining the appropriate SRS methodology it is important to consider the end-goal of the survey: population estimates, vaccination coverage, or both. The photo-based method is time-intensive, which should factor into this consideration. When determining vaccination coverage after a vaccination campaign one must carefully consider the method and quality of dog marking, as we have shown that even with a 2-mark system, small delays in evaluation can lead to significant under-estimations of vaccination coverage. The photo-based method of SRS has proven to be a valuable tool for comparison and validation, providing insight into how best to evaluate dog vaccination campaigns.

## Disclosure

The views expressed in this publication are those of the authors and may not reflect the views or policies of the U.S. Government.

## Declaration of Competing Interest

None.
