# Supplementary material for: Use of photography to identify free-roaming dogs during sight-resight surveys: Impacts on estimates of population size and vaccination coverage, Haiti 2016
Source: Vaccine X. 2019 Jun 19;2:100025. doi: 10.1016/j.jvacx.2019.100025 (PMC6668240; doi:10.1016/j.jvacx.2019.100025)
Supplement: Supplementary data 1 [file mmc1.docx]

Appendix I.

**Haiti SRS Dog Population Data Capture Form**

SITE ID: ____________ DATE: __ / __ / ____ NAME: ________________ RESIDENTIAL? Y / N

HUMAN POPULATION: ____________ WEATHER CONDITIONS: ____________ Start Time: __________ End Time: __________

| **Unique Dog ID** | **GPS Mark Number** | **Identification** | ***Description of Dog** | ****Sex** | **BCS**  **1 - 9** | **Age** | **Sight or Resight?** | **Confinement** | **Visible Fresh Wounds** | **Visible Healed Wounds** |
| --- | --- | --- | --- | --- | --- | --- | --- | --- | --- | --- |
|  |  | Vax ID: _________  Vax Collar? Y / N  Other Collar? Y / N | Size? S M L  Color?  Distinct Markings: | MI  MN  F  FL |  | Puppy  Adult |  | Running loose  Walking on leash  Tied on property  Behind wall  Other: |  |  |
|  |  | Vax ID: _________  Vax Collar? Y / N  Other Collar? Y / N | Size? S M L  Color?  Distinct Markings: | MI  MN  F  FL |  | Puppy  Adult |  | Running loose  Walking on leash  Tied on property  Behind wall  Other: |  |  |
|  |  | Vax ID: _________  Vax Collar? Y / N  Other Collar? Y / N | Size? S M L  Color?  Distinct Markings: | MI  MN  F  FL |  | Puppy  Adult |  | Running loose  Walking on leash  Tied on property  Behind wall  Other: |  |  |
|  |  | Vax ID: _________  Vax Collar? Y / N  Other Collar? Y / N | Size? S M L  Color?  Distinct Markings: | MI  MN  F  FL |  | Puppy  Adult |  | Running loose  Walking on leash  Tied on property  Behind wall  Other: |  |  |
